# Mesoporous crystalline Ti_1-x_Sn_x_O_2_ (0 < x < 1) solid solution for a high-performance photocatalyst under visible light irradiation

**DOI:** 10.3389/fchem.2022.1111435

**Published:** 2022-12-14

**Authors:** Wen Yu, Nankun Geng, Jianming Han, Wenjun Yu, Yiting Peng

**Affiliations:** Shanghai Key Laboratory of Materials Protection and Advanced Materials in Electric Power, Shanghai University of Electric Power, Shanghai, China

**Keywords:** TiO2, solid solution, aerosol-spray, self-assembly, photocatalyst

## Abstract

We report a facile and effective inorganic polycondensation combined with aerosol-spray strategy towards high-performance photocatalyst by fabricating mesoporous Ti_1-x_Sn_x_O_2_ (0 < x < 1) solid solution. Such Ti_1-x_Sn_x_O_2_ nanocrystals with high Sn-doped contents are self-assembled into mesoporous spheres can effectively promote visible-light harvest and high quantum yield, leading a longer lifetime of the photoelectron-hole pairs and less recombination. Such the photocatalysts enhanced photocatalytic activity for the degradation of Rhodamine B (RhB). The representative Ti_0.9_Sn_0.1_O_2_ and Ti_0.8_Sn_0.2_O_2_ compounds reach an optimum degradation of ≈50% and 70%, respectively, after 120 min irradiation under visible irradiation. The mesoporous Ti_1-x_Sn_x_O_2_ solid solution could inhibit the recombination of electron-hole pairs, which promote reaction thermodynamics and kinetics for RhB degradation.

## 1 Introduction

Rapid development and the transition to a carbon-neutral society have highlighted the need for photovoltaic cells to use high-efficiency photocatalysts under visible light (>380 nm) (Liu et al., 2016; Galvao et al., 2018; González-Gutiérrez et al., 2019). Transition metal oxides (such as TiO_2_ and SnO_2_) have attracted considerable attention from researchers due to their excellent photocatalytic activity, high natural abundance, and cost-effectiveness (Sharon et al., 2016). Owing to two oxides with different band-gap energies (*E*_g_) of 3.2 eV for TiO_2_ and 3.8 eV for SnO_2_ (Nuansing et al., 2006), respectively, the enhanced charge separation occurs in their mixture where electrons are injected from the conduction band of SnO_2_ to the valence band of TiO_2_, leading to better performance than individual semiconductor oxides due to their synergistic effects. To better regulate their band gap and obtain high efficiency, more efforts should be focused on building a desired heterostructure and tuning electron correlation by varying the stoichiometry of oxides Luo et al. (2018). Heterostructures were mainly fabricated by the assembly of two or more oxides that were physically (e.g., Van der Wall’s force) or chemically bonded together, exhibiting superiority of geometry and junction interface (Li et al., 2021; Liu et al., 2023). For heterostructure-based metal oxide, element doping coupled with hierarchy design achieved visible benefits due to the formation of a single phase rather than a combination of two oxide compounds. For example, the pre-synthesized SnO_2_ quantum dots directed growth on titania show slow charge transfer, low active-sensitizer loading and poor uniformity control for deposition on high aspect-ratio TiO_2_ nanostructures (Wang et al., 2022). Doping titania with either inorganic or metallic species can generate donor or acceptor states in the band gap of TiO_2_ and impurity states that enhance visible-light adsorption (Albornoz et al., 2021; Wu et al., 2022). Non-etheless, metal oxides with doping heterostructure often suffered from thermal instability, fast recombination rates, or the requirement of an expensive ion-implantation facility for more elaborate control, and even showed much larger formation energy due to the substitution of large-radius metal ions (Singh et al., 2021; Liu et al., 2022). Many essential questions remain, such as the effect of the structure-function correction, composition, and interfaces on the photocatalytic activity, which remains challenging.

One promising strategy to obtain a heterostructure-based titania photocatalyst is to fabricate a substitutional solid solution using metal atoms with a comparable ionic radius (VI-coordinated) with titania (Reddy et al., 2008). Such a metal-doped TiO_2_ solid not only retains the benefits from the quantum structures of crystalline titania during photocatalytic processes but further enhances photoinduced surface redox reactions one. Compared with the other cationic doping (such as Cr, V, and Fe), Sn ion shows a more favorable effect on photocatalytic activity in water-splitting and oxidation of organic compounds (Liang et al., 2012). To date, few examples of tin-doping titania (denoted as Ti_1-x_Sn_x_O_2_, 0 < x < 1) have been reported, especially on nanoscale systems (Asokan et al., 2010). Such compounds have been generally synthesized by solid-state reaction at high temperatures beyond 1,200°C or chemical deposition technology, such as hydrothermal (Chen et al., 2022; Zhao et al., 2016; Ruiz Esquius et al., 2020), sol-gel (Pudukudy et al., 2017; Liu et al., 2019), electrospinning (Yu et al., 2018), and polyol-mediated methods (Pelicano et al., 2022). However, these methods are limited because of the difficulty in elaborate control of doping contents and nanoparticle aggregation and the incapability of constructing hierarchical pore structures, leading to an increased probability of recombination. Besides, most compounds prepared using the above-mentioned methods have a rutile tetragonal crystalline structure with no anatase analog due to the existence of Sn (Qin et al., 2021). The latter has been proposed to have higher photoactivity than the former, but it is still limited by a less Sn-doping content (<0.3).

**FIGURE 1:**
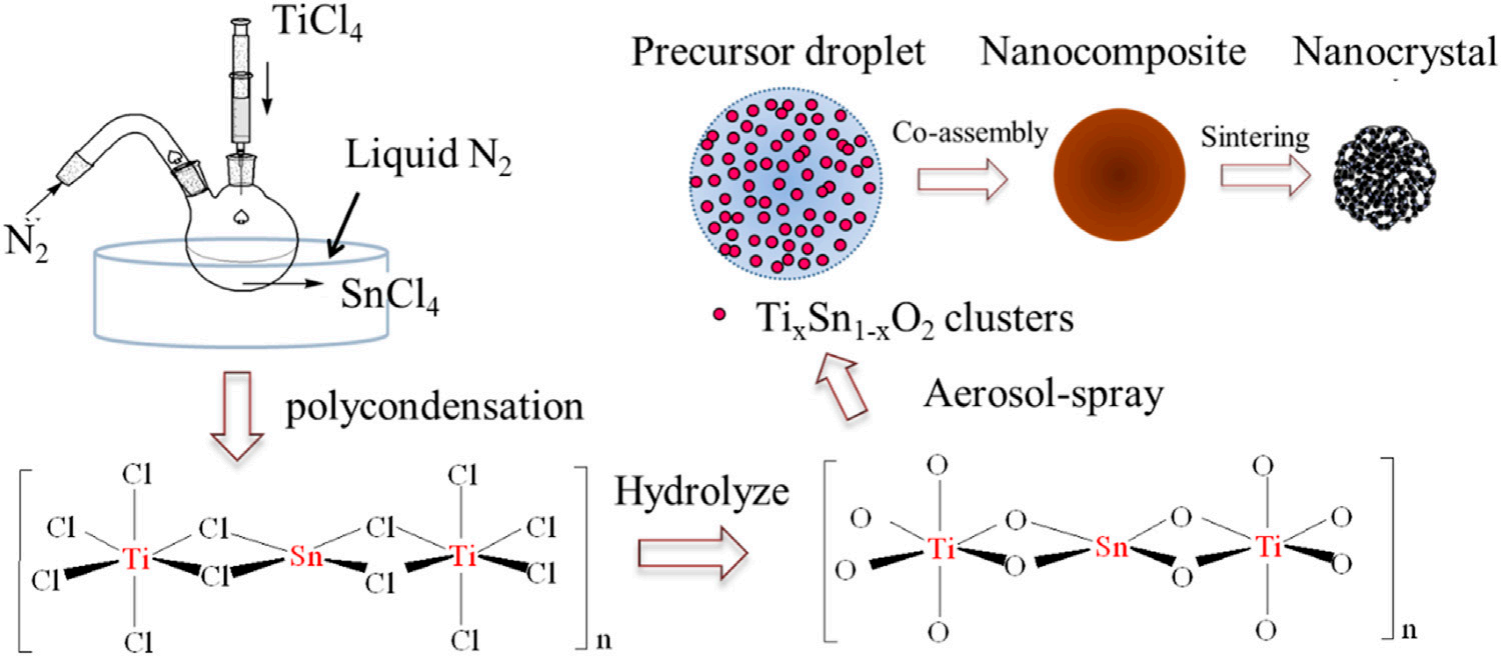
Schematic representation of the mesoporous Ti_1-x_Sn_x_O_2_ (0 ≤ x ≤ 1) nanocomposites.

To address this challenge, we report a facile and effective doping method to prepare a hierarchically mesoporous Ti_1-x_Sn_x_O_2_ compound for high-performance photocatalyst under visible light irradiation. Inspired by the Ziegler-Natta catalyst mechanism, Figure 1 illustrates an inorganic polycondensation process that occurs between titanium chloride and tin chloride liquid in a liquid nitrogen bath. The ultra-low temperature effectively suppresses chloride salt hydrolysis and causes chloride ions from tin chloride to fill the empty orbital of a 6-coordinated Ti atom. Then, Ti atoms and Sn atoms were bridged to two chlorine atoms to obtain successive -Ti-Cl-Sn-Cl-Ti- chains, allowing for high Sn-contents. Subsequently, the mesoporous Ti_1-x_Sn_x_O_2_ spheres are synthesized using a low-cost aerosol-spray method. Ti_1-x_Sn_x_O_2_ nanocrystals generated from such a preformed aqueous solution are self-assembled into mesoporous spheres. Such a hierarchical heterostructure endows several advantages to the Ti_1-x_Sn_x_O_2_ compound. Firstly, mesoporous Ti_1-x_Sn_x_O_2_ spheres inherit the anatase crystalline structure from titania and small nanocrystal size, which achieves a high surface area and many active sites. Second, hierarchically porous architecture coupled with high Sn-contents can effectively promote visible-light harvest and high quantum yield. Finally, the mesoporous structure throughout the spheres endows effective transport of charge carriers, allowing for longer lifetimes of the photoelectron-hole pairs and less recombination. Such a strategy could be easily extended to other photocatalyst designs and large-scale fabrication.

## 2 Experiment section

### 2.1 Materials preparation

Pluronic surfactant F127 (EO106PO70EO106), titanium chloride (TiCl_4_; ≥98%), and tin (IV) chloride (SnCl_4_, ≥99%) were purchased from Sigma-Aldrich Corp. All chemicals were used without further purification. The Ti_1-x_Sn_x_O_2_ (0 < x < 1) solid solutions and the undoped TiO_2_, SnO_2_ were all synthesized by an aerosol-spraying process using TiCl_4_, SnCl_4_ and tri-block copolymer F127 aqueous solution as a precursor. Such a stable and homogeneous precursor was achieved by a polycondensation process using various mole ratios (Ti: Sn = 1-x: x (0 < x < 1) under liquid nitrogen for 2 h and then slowly injecting 100 ml of F127 (0.5 g) aqueous solution. The mole fraction of Sn-containing precursor in the mixture is denoted by *x* = mmol Sn/(mmol Ti + mmol Sn). The mixture solution was vigorously stirred for 30 min and subsequently used an aerosol-assisted self-assembly process to continuously generate droplets from the precursors. The droplets were then sent through a furnace and reacted at 450°C to form homogenous particles. Calcination was carried out in a box furnace at 350°C for 3 h and then 550°C for another 3 h under the air atmosphere. The heating rate for the whole process was kept at 2°C·min^−1^. As-made products were milled and grounded into fine powder.

### 2.2 Material characterization and measurement

X-ray diffraction (XRD) patterns were taken on a Panalytical X'Pert Pro X-ray powder diffractometer powders diffractometer using copper *Kα* radiation (*λ* = 1.54 Å). Nitrogen sorption isotherms were measured at 77 K with a Micromeritics ASAP 2020 analyzer. The samples were degassed in vacuum at 180°C for 3 h before measurements were taken. The specific surface areas were calculated by the Brunauer–Emmett–Teller (BET) method using the adsorption branch in the relative pressure range from 0.04 to 0.25. The pore size distributions (*D*_p_) were derived from the adsorption branch using the Barrett–Joyner–Halenda (BJH) model. Scanning electron microscopy (SEM) images and energy-dispersive spectroscopy (EDX) were obtained using a JEOL JSM-6700 FE-SEM at 200 kV to obtain morphology and elemental information of the products. Transmission electron microscopy (TEM) images were obtained using a Philips CM120 microscope operated at 120 kV. Surface composition was analyzed by X-ray photoelectron spectroscopy (XPS). Core level photoemission spectra of C 1 s and O 1 s lines were collected with a PHI 3057 spectrometer using Mg *K*α X-rays at 1,286.6 eV. All XPS spectra were taken in small area mode with an acceptance angle of 78° and 23.5 eV pass energy. All spectra were referenced to the C 1 s peak of the graphitic carbon atom at 285.0 eV. The integrated areas of the Ti 2*p* and Sn 3 days photoemission peaks, divided by their sensitivity factors 0.665/1.334 and 3.286/4.725, were used to determine surface atomic percentages. UV-vis absorption spectra were recorded on a Shimadzu UV-1700 spectrometer with a resolution of 2.0 nm.

### 2.3 Photocatalytic activity measurement

Under visible light irradiation, the photocatalytic activities of the Ti_1-x_Sn_x_O_2_ (0 < x < 1) solutions were evaluated by the photodegradation of organic contaminants, and Rhodamine B (RhB, M_w_: 479.02) was used as the model pollutant. A set of photocatalytic activity experiments on samples were performed with the following procedures: 0.1 g of photocatalyst was put into 30 ml of DI-water and stirred at room temperature for 1 h to make the photocatalyst powder uniformly dispersed. Then RhB was added into the suspension to keep a concentration of 10^−5^ mol·L^−1^. Then the mixture was stirred in the dark for 30 min to reach adsorption–desorption equilibrium. From the top, the suspension was vertically irradiated with simulated visible light from a 500 W Xe lamp equipped with a UV cutoff filter (>400 nm) with distance of 15 cm between the lamp and the suspension surface. During the photo reaction, the samples were collected at different time intervals; 0 min, 10 min, 30 min, 60 min, 90 min, and 120 min for analysis. In order to estimate the photocatalytic stability, a cycle photocatalytic degradation experiment was conducted as follows: the original concentration of RhB dye in the reactor was tuned to 10^−5^ mol·L^−1^ every 120 min as one cycle, and then the samples were picked up at an interval of 45 min for analysis. The concentration of the RhB was determined by monitoring the height of the maximum (*λ* = 554) of the absorption in UV-vis spectra.

## 3 Results and discussion

**FIGURE 2:**
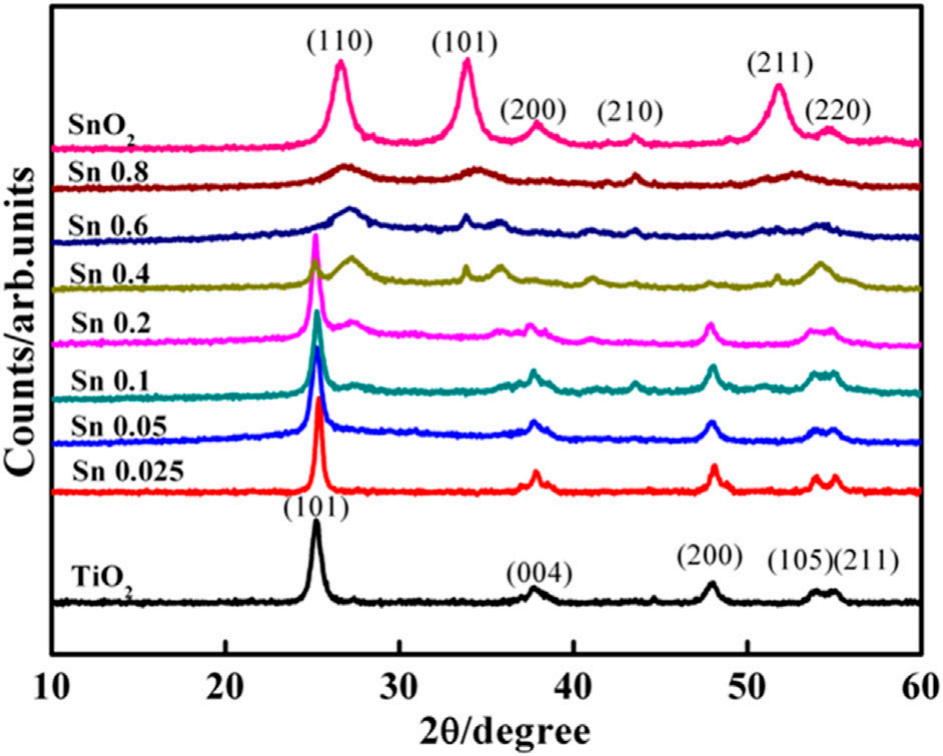
XRD curves of the TiO_2_, Ti_1-x_Sn_x_O_2_ and SnO_2_ nanocrystals prepared by aerosol method.

Figure 2 compares the crystallinity of as-synthesized Ti_1-x_Sn_x_O_2_ nanocrystals with various Sn-doped contents. The diffraction pattern of as-synthesized TiO_2_ nanocrystals match well to that of anatase structure with intense (101) (004), and (200) reflections, corresponding to the 25.4°, 37.8° and 48.1° 2θ angles (JCPD No. 04-0477). Consistently, as-synthesized SnO_2_ nanocrystals show crystallized rutile structure with well-defined peaks at 25.3° and 27.4°corresponding to (101) and (110) plane (JPCD No. 84-1286), respectively. Upon increasing Sn-doped, the reflection of Ti_1-x_Sn_x_O_2_ compounds (x ≤ 0.05) almost retain the anatase phase but a shift toward lower 2θ angels (∼0.1°) at (101) peak position. While Ti_1-x_Sn_x_O_2_ (0.05 < x ≤ 0.4) exhibit two crystalline structures with strong anatase and weak rutile, indicative of crystalline phase transition. For Ti_1-x_Sn_x_O_2_ (0.6 < x ≤ 0.8), their characteristic peak positions are consistent with rutile SnO_2_ (110) peaks at 26.6° and anatase TiO_2_ reflection gradually disappeared.

**FIGURE 3:**
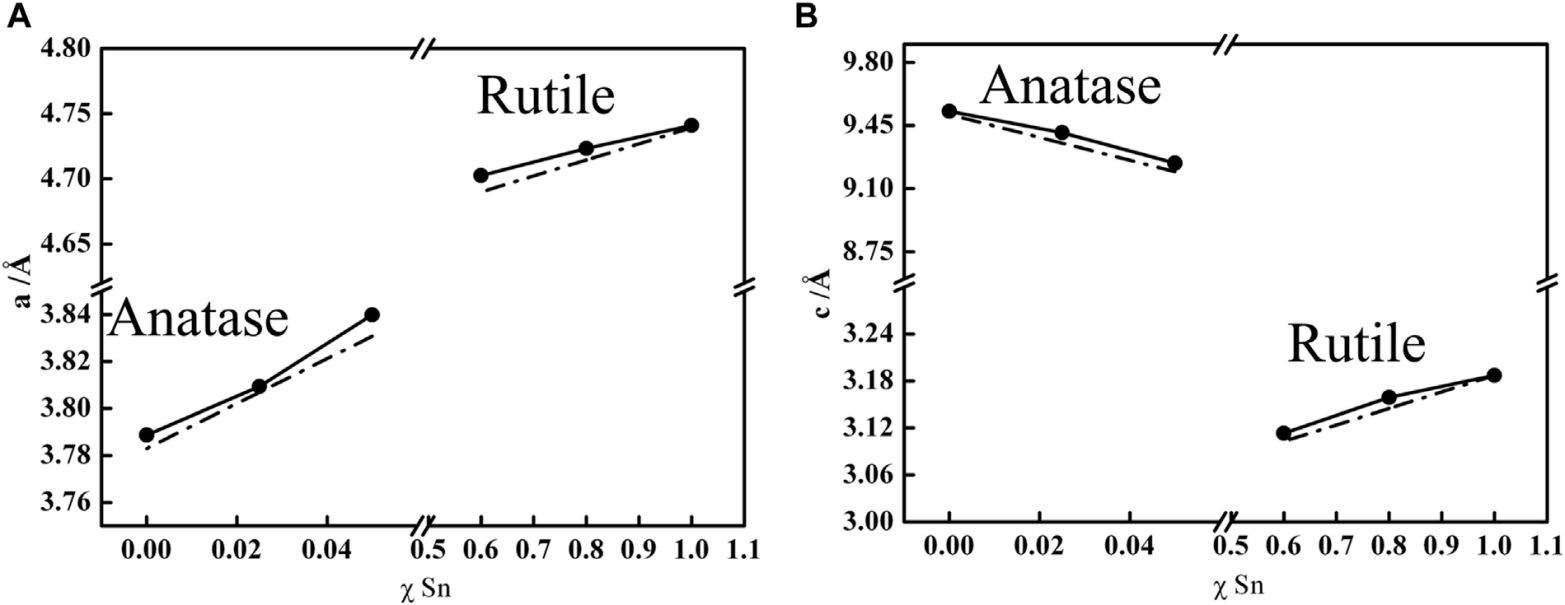
Dependence of the mole fraction of Sn (χ) on the *a* **(A)** and *c* **(B)** lattice parameters of Ti_1-x_Sn_x_O_2_ (0 < x < 1) solid solution (solid lines). Dash dot lines represent the theoretical values according to Vegard’s law calculation.

To further confirm the Ti_1-x_Sn_x_O_2_ formed in a single crystalline phase, Figure 3 compares experimental lattice parameters (*a*, *c*) and unit cell volume (*V* = *ac* ^2^) of the anatase Ti_1-x_Sn_x_O_2_ (*x* ≤ 0.05) and the rutile Ti_1-x_Sn_x_O_2_ (0.5 < *x* ≤ 0.8) according to Vegard’s law (Kong et al., 2019). The Vegard’s law is an approximate empirical rule that predicts the relationship between lattice constant and the concentration of the constituent elements solid solution, as describe as:

$$a=x_{1}a_{1}+\left(1-x_{1}\right)a_{2}$$ (1)

where *a* is the lattice parameter for the solid solution, *a*_1_ and *a*_2_ are lattice parameters of individual oxide, and *x*_1_ is the mole fraction of metal ions. Figure 3A shows the linear trend of lattice parameters *a* for the Ti_1-x_Sn_x_O_2_ compounds, indicating that the hetero-Sn atom-doped into the TiO_2_ lattice brings the lattice expansion. Similarly, the lattice parameter *c* of rutile Ti_1-x_Sn_x_O_2_ with high Sn-contents (0.5 < x ≤ 0.8) retains the identical trend, as shown in Figure 3B. In contrast, the lattice parameters *c* of anatase Ti_1-x_Sn_x_O_2_ with low concentration of Sn-contents shows linearly decrease, implying large anisotropy compressibility occurs throughout anatase TiO_2_ crystal. The different compressibility (*κ*, *κ*_a_ > *κ*_c_) causes more denser cell lattice with increasing Sn contents, which is consistent with impact of the external stress on solid solution structure (Prasad and Mikula, 2000). Additionally, the phase separation may cause certain positive deviate on due to spinodal deposition. Table 1 summarized the anatase-rutile phase distribution of TiO_2_, Ti_1-x_Sn_x_O_2_ (0 < x < 1), and SnO_2_ according to calculation of the lattice parameters and mass fraction of rutile phase.

$${Sn}^{2+}+O_{2}+2H_{2}O+2e^{-}\to {Sn}^{4+}+4{OH}^{-}$$ (2)

where *W*_R_ is the mass fraction of rutile phase, *I*_R_ is correction factor which indicates the difference of scattering intensities between crystalline structures, and *I*_A_ and *I*_R_ represent the integrated intensities of the anatase (101) and rutile (110) reflection, respectively. The mass fraction of rutile phase continuously increases from 14.8 wt% for Ti_0.9_Sn_0.1_O_2_ to 60.6 wt% for Ti_0.4_Sn_0.1_O_2_. Indeed, Sn atoms in TiO_2_ lattices could provide large number of dissimilar boundaries, which would suppress the crystal growth of TiO_2_ in the dealloying process, finally leading to extremely fine nanostructure in TiO_2_-SnO_2_ solid solution (Sayılkan et al., 2008; Wu et al., 2017). Hence, such a series of Ti_1-x_Sn_x_O_2_ (0 < x < 1) compounds underwent pure anatase, mixed anatase-rutile to pure rutile phases, respectively, which provide a fundamental understanding of the relationship between electronic structure and dye-sensitized photocatalytic degradation.

**TABLE 1:** The summary of lattice parameters of (101), rutile (110) and (101) plane from anatase lattice; and mass fraction of rutile phase (W_R_).

Sample	Peak position (2θ)			Mass fraction of rutile (wt%) W_R_
	A (101)	R (110)	R (101)	
TiO_2_	25.3°			0.0
Ti_0.975_Sn_0.025_O_2_	25.3°			0.0
Ti_0.95_Sn_0.05_O_2_	25.2°			0.0
Ti_0.9_Sn_0.1_O_2_	25.2^o^	27.4°		14.8
Ti_0.8_Sn_0.2_O_2_	25.2°	27.3°		19.7
Ti_0.6_Sn_0.4_O_2_	25.1°	27.4°	35.8°	60.6
Ti_0.4_Sn_0.6_O_2_		27.1°	34.8°	100
Ti_0.2_Sn_0.8_O_2_		26.9°	34.5°	100
SnO_2_		26.6°	33.9°	100

**FIGURE 4:**
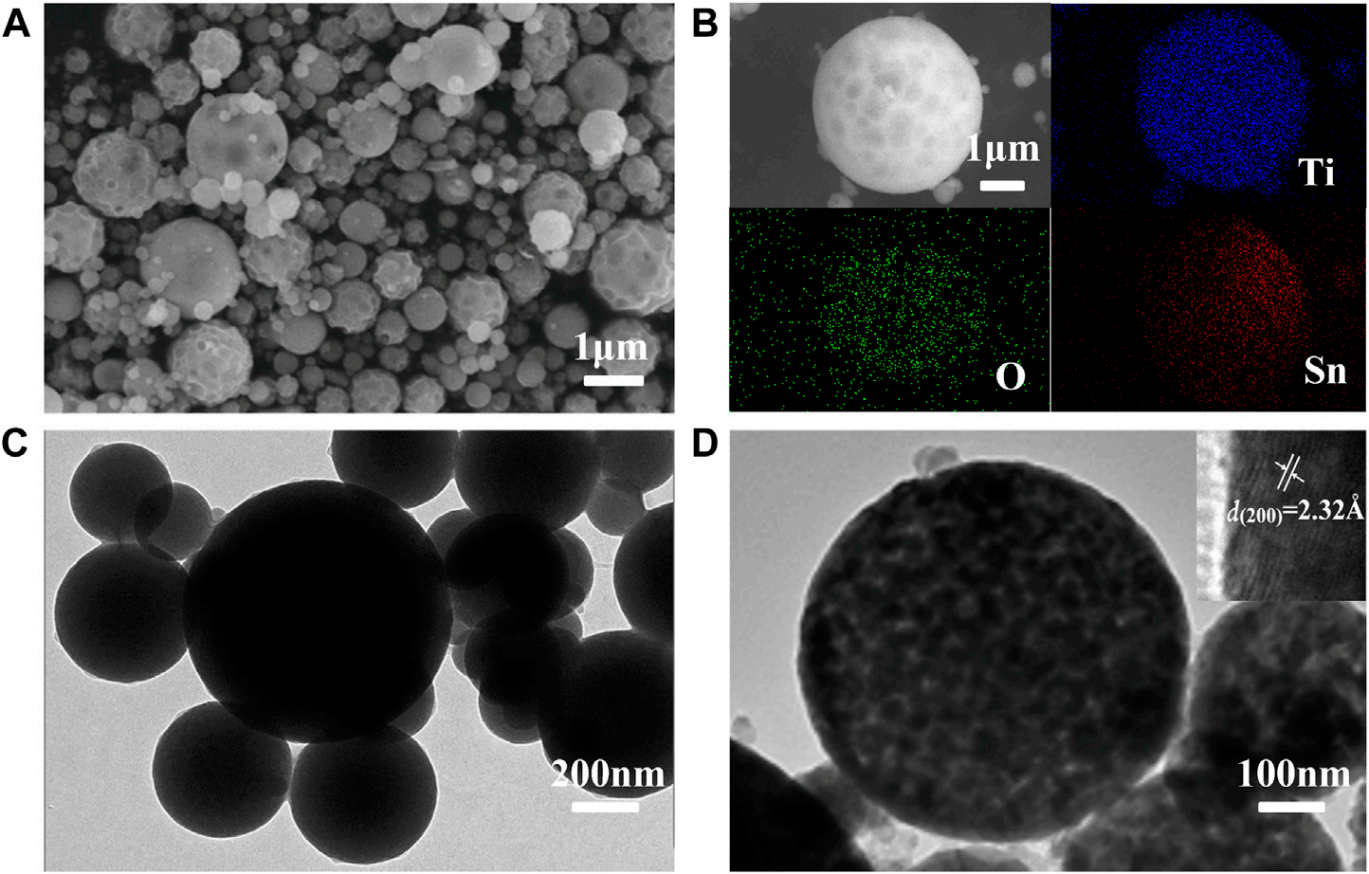
**(A,B)** SEM and elemental mapping images and **(C,D)** TEM images of the Ti_0.8_Sn_0.2_O_2_ solid-solution after sintering at 550°C for 3 h.

The morphology and elements analysis of Ti_1-x_Sn_x_O_2_ (0 < x < 1) compounds obtained after self-assembly are characterized by scanning electron microscope (SEM) combined with energy-dispersive X-ray (EDX), as shown in Figures 4A,B. All samples show similarly spherical morphology with a diameter range from 150 to 500 nm, exemplified by Ti_0.8_Sn_0.2_O_2_. The bestrewed pot-holes on the spheres surface are also observed arising from the stacking of hard-sphere crystals. The uniform distribution of each element suggests that the presence of Ti and Sn atoms throughout crystalline structure. Transmission electron microscopic (TEM) image clearly displays the as-sprayed Ti_0.8_Sn_0.2_O_2_ particles with non-porous spherical morphology before sintering (Figure 4C). After sintering, the compounds become highly porous due to removal of surfactant, and each sphere is composed of uniform nanocrystals with diameter around 5–6 nm. High-resolution TEM (HRTEM) in Figure 4D shows the ultrafine nanocrystals with an interlayer spacing of 2.32 Å, corresponding to the (200) plane of rutile TiO_2_ (∼2.29 Å). This result is consistent with the XRD patterns, showing initial extension of *c* axis occurs during the anatase-rutile conversion at higher Sn-contents doping. Such hierarchically porous design and highly crystalline structure can provide high surface area and fast kinetics, which favors the transport of charge carriers and prevent the recombination of photoelectron-hole pairs.

**FIGURE 5:**
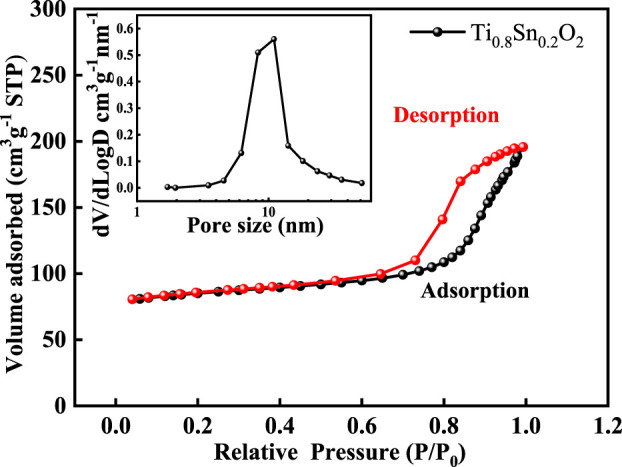
Nitrogen sorption isotherms and pore size distribution (inset) of the Ti_0.8_Sn_0.2_O_2_ compound.

Nitrogen sorption experiment was further conducted to probe the pore structure. Figure 5 shows the nitrogen sorption isotherms and pore size distribution of anatase Ti_0.8_Sn_0.2_O_2_ solid solution with rutile-phased fraction of 20 wt%, confirming a hierarchical pore structure including micropore, mesopore, and macropores arising from particles aggregation. The significant nitrogen uptake at the relative pressures at 0.4–0.6 is consistent with a mesopore network with a narrow pore distribution of ∼10 nm (inset). Table 2 summarizes the surface area and pore structure parameters of Ti_1-x_Sn_x_O_2_ (0 < x ≤ 0.4). Upon increasing the Sn-doped contents, the surface areas of Ti_1-x_Sn_x_O_2_ compounds shows significantly decrease from 77.58 m^2^·g^−1^–44.96 m^2^·g^−1^ due to introduction of heavier Sn atoms. Additionally, the average pore volumes of Ti_1-x_Sn_x_O_2_ maintain approximately ∼0.15 cm^2^·g^−1^.

**TABLE 2:** The parameters of pore structure in pure TiO_2_ and Ti_1-x_Sn_x_O_2_ (0 < x ≤ 0.4).

Sample	Surface area/m^2^·g^−1^	Average pore size/nm	Pore volume/cm^3^·g^−1^
TiO_2_	77.58	8.75	0.18
Ti_0.95_Sn_0.05_O_2_	57.28	14.58	0.16
Ti_0.9_Sn_0.1_O_2_	56.00	13.77	0.14
Ti_0.8_Sn_0.2_O_2_	50.06	10.67	0.19
Ti_0.6_Sn_0.4_O_2_	44.96	8.42	0.21

**FIGURE 6:**
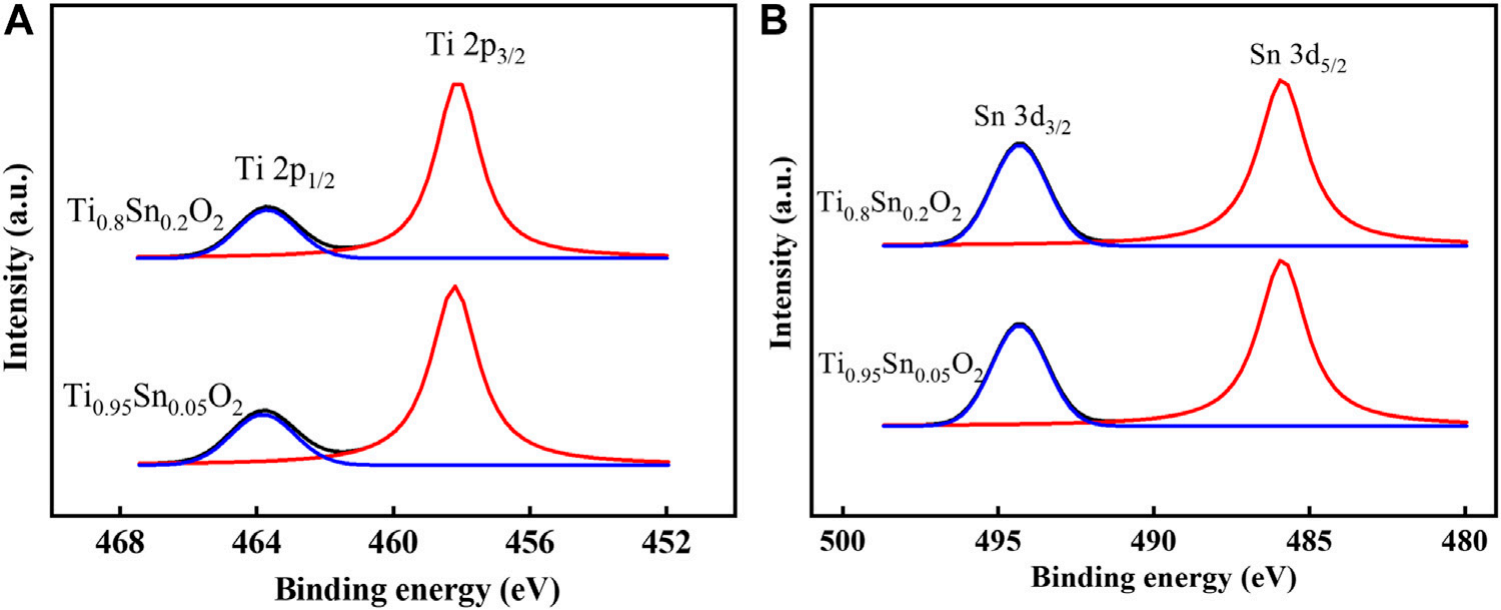
XPS curves fitted of **(A)** Ti 2p spectra of Ti_0.95_Sn_0.05_O_2_ and Ti_0.8_Sn_0.2_O_2_, **(B)** Sn 3*d* spectra of Ti_0.95_Sn_0.05_O_2_ and Ti_0.8_Sn_0.2_O_2_.

To further understand the electronic properties depended transformation between anatase and rutile phase, the elemental composition and valence state in anatase Ti_0.95_Sn_0.05_O_2_ and anatase-rutile Ti_0.8_Sn_0.2_O_2_ are conducted by X-ray photoelectron spectra (XPS), as shown in Figure 6. Figure 6A shows the Ti 2*p* fitting curves for, which presents two Ti 2*p* peaks at *E* = 458.2 eV and *E* = 463.7 eV for Ti 2*p*_3/2_ and Ti 2*p*_1/2_ lines, respectively. Which is consistent with the values of Ti^4+^ reported previously (Fan et al., 2021). Meanwhile, Figure 6B represents typical Sn 3*d* curves for Ti_0.95_Sn_0.05_O_2_ and Ti_0.8_Sn_0.2_O_2_ with both peaks at 494.2 eV and 485.9 eV for Sn 3*d*_3/2_ and Sn 3*d*_5/2_ lines, interpolating peak positions of the metallic Sn and SnO_2_ at 493.1/495.0 eV and 484.7/486.4 eV for Sn 3*d*_3/2_/Sn 3*d*_5/2_ lines, which can be ascribed to the Sn^4+^ species. These results indicate no changes in valence state of Sn and Ti during phase transformation, implying the distorted degree in tetragonal structure is neglected at low Sn concentration. Together with XPS analysis, Table 3 compares the atomic concentration ratios of O 1 *s* and Ti 2*p* for undoped TiO_2_, Ti_0.95_Sn_0.05_O_2_ and Ti_0.8_Sn_0.2_O_2_. The concentration ratio of Ti 2*p* to O 1 *s* decrease with increasing Sn contents, suggesting the Sn-doped in the tetragonal TiO_2_ lattice for formation of Ti-O-Sn bonding.

**TABLE 3:** States of Ti_0.95_Sn_0.05_O_2_ and Ti_0.8_Sn_0.2_O_2_ solid solution and elemental surface composition determined by XPS.

Sample		TiO_2_	Ti_0.95_Sn_0.05_O_2_	Ti_0.8_Sn_0.2_O_2_
BE (eV)	O 1 s	530.3	529.5	529.4
	Ti 2p3/2	459.8	458.2	458.2
	Sn 3d5/2	—	485.9	485.9
Atomic Conc (%)	O 1s	47.3	46.6	45.8
	Ti 2p3/2	17.2	15.3	8.7
	Sn 3d5/2	—	0.8	2.0

**FIGURE 7:**
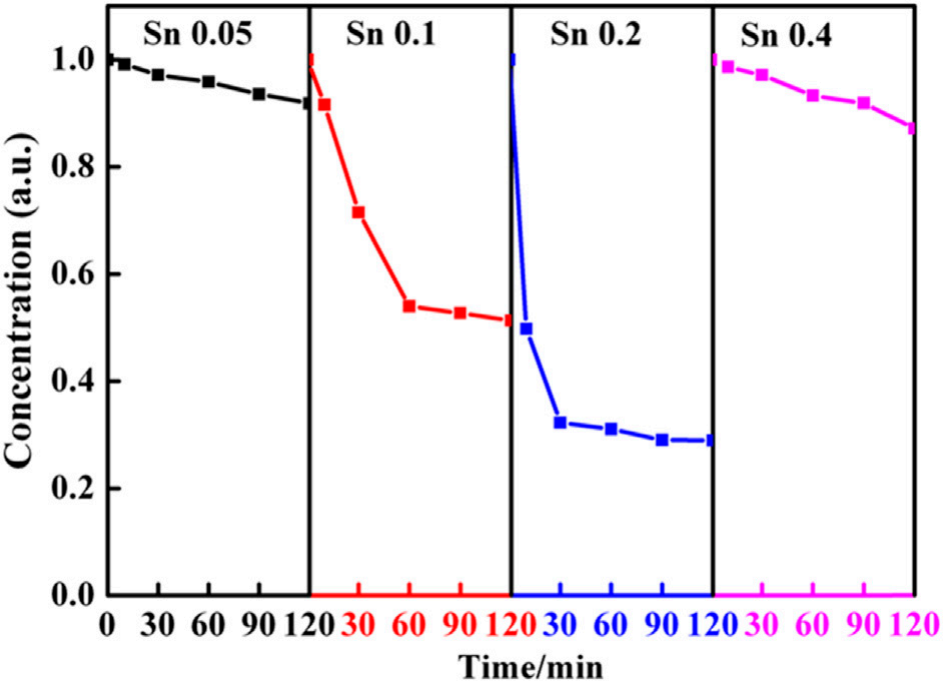
Dependence of the Sn-doped contents on the reaction time of Ti_1-x_Sn_x_O_2_ (0.05 ≤ x ≤ 0.4) compounds with RhB under visible-light harvester.

To demonstrate this intricate structure-function correction, we evaluate the photoactivity of the porous Ti_1-x_Sn_x_O_2_ (0.05 ≤ x ≤ 0.4) nanoparticles in degradation of RhB dye under visible light irradiation, as shown in Figure 7. The blank experiment showed that the concentration of RhB solution without catalyst maintain during 90 min, indicating the RhB molecules are relatively stable and hard to decompose. Before photocatalytic degradation, it is necessary to the eliminate an adsorption effect by stirring the mixtures in the dark for 1 h. Then the Ti_1-x_Sn_x_O_2_ compounds were added in RhB solution being treated in the dark for another 60 min. All the samples have strong physical adsorption abilities, but porous solid solution can enhance adsorption ability than the blank samples. After adsorption, the next photocatalytic degradation experiments kept the RhB concentration at equilibrium as the starting point. Upon increasing Sn-doped contents, the Ti_0.9_Sn_0.1_O_2_ and Ti_0.8_Sn_0.2_O_2_ solutions with mixed anatase-rutile phases exhibit the higher photoactivity (RhB degradation yield ≈50% and 70%, respectively after irradiation for 120 min) than that of the anatase Ti_0.95_Sn_0.05_O_2_ solution (RhB degradation yield <10% after irradiation for 120 min). However, the photocatalytic activity of Ti_0.6_Sn_0.4_O_2_ with higher rutile phased of 60 wt% decrease with increasing Sn content because the RhB degradation yield is only about 15% after irradiation for 120 min.

**FIGURE 8:**
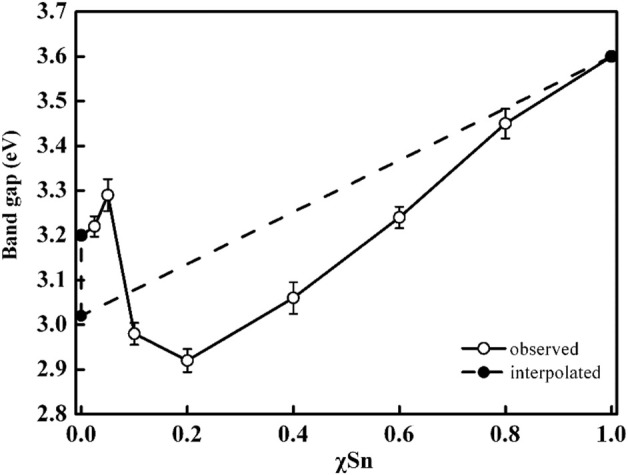
Variation in optical band gap of Ti_1-x_Sn_x_O_2_ (0 < x < 1) samples. Dashed line is a linear interpolation between the band gap values for anatase TiO_2_ (3.2 eV), rutile TiO_2_ (3.0 eV), and SnO_2_ (3.60 eV).

In the heterojunction composed of TiO_2_ and SnO_2_, the photogenerated electrons can transfer from TiO_2_ to SnO_2_ phase with no applied voltage (Fan et al., 2016). Because of their identical rutile phase, tetragonal crystal structures, ionic radius (0.690 for Sn^4+^ and 0.605 for Ti^4+^), and electronic characteristics, SnO_2_ and TiO_2_ may also form solid solutions coupling system (Sayılkan, 2007; Huang et al., 2015). Thus, it is very essential to probe the relationship between electronic structure and photoactivity in Ti_x_Sn_1-x_O_2_ solutions. Figure 8 illustrates the dependence of band gap of Ti_1-x_Sn_x_O_2_ (0 ≤ x ≤ 1) with increasing Sn-doping. Theoretical considerations by Long et al. suggest that for substitutionally Sn-doped anatase TiO_2_, the band gap increases ∼0.13 eV owing to high formation energy while maintain anatase phase (Long et al., 2009). However, the substitution of Sn for rutile TiO_2_ brings a red-shift of the adsorption edge deriving from the decrease of Sn 5s gap state in the conduction band (Chang et al., 2019). Consistent with the theoretical analysis, the experimental band gaps of the anatase Ti_1-x_Sn_x_O_2_ (x = 0.05 and 0.025, respectively) solid solutions indeed increase to 3.22 and 3.29 eV, respectively (Ren et al., 2020). High Sn-doped contents causes a transition from anatase to rutile phase, and the band gap of solution with mixed phase should be reasonably neutralized in between that of single phase using their mass fraction (Chen et al., 2010; Mohammad et al., 2021). However, it is worth noting that the band gap values of Ti_0.9_Sn_0.1_O_2_ and Ti_0.8_Sn_0.2_O_2_ decreases to 2.98 and 2.92 eV, respectively. This is possibly attributed to transition of photoexcited electrons and holes from rutile to energy states of anatase phase, which needs very low energy about 0.2∼0.25 eV. Meanwhile, we discovered that the presence of crystalline interface within mixed phases effectively reduces electron-hole recombination and creates more oxygen vacancies to enhance visible-light harvesting ability.

**FIGURE 9:**
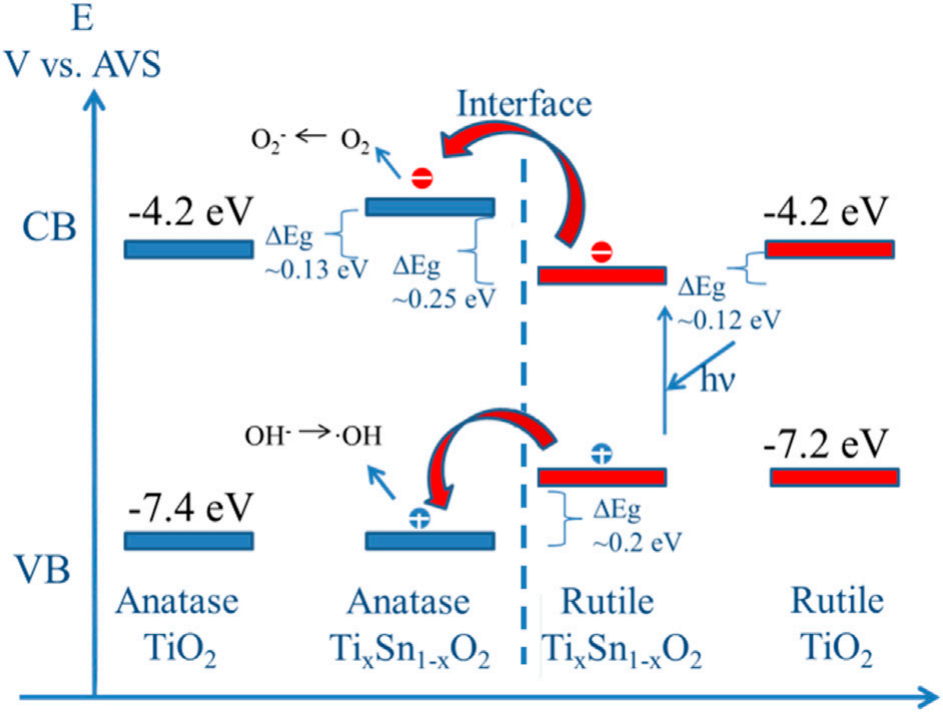
Scheme of electron transition between mixed Ti_1-x_Sn_x_O_2_ (0.1 ≤ x ≤ 0.4) solutions. The absolute valence band and conduction band edges relative to vacuum level of TiO_2_ are obtained from the reference.

Continuation of this process gradually separates photoexcited electron-hole pairs, sequentially providing more photo-charges needed for the photocatalytic reaction and fast charge migration, as shown in Scheme of Figure 9. The rapid increased band gap values of Ti_1-x_Sn_x_O_2_ (0.4 ≤ x ≤ 0.8) is attributed to the more Sn doping, leading the conduction band to shift toward a higher energy, which accounts for the observed decreasing photocatalytic activity at a higher Sn content (e.g., x ≥ 0.8). Rather than facilitate charge migration and reduce charge recombination, the charge trapping may take effect as centers for electro-hole recombination and absorb the visible light, described as follow reaction 1 and 2 (Golestanbagh et al., 2018; Shi et al., 2007):

$${Ti}^{4+}+e^{-}\to {Ti}^{3+}$$ (1a)

$${Sn}^{4+}+2e^{-}\to {Sn}^{2+}$$ (2a)

$${O^{2}}^{-}\to O+V_{o}+2e^{-}$$ (3)

The visible-spectra sensitization of Sn-doped TiO_2_ is attribute from charge transfer bands of peroxotitanium or the formation of Sn II) with stable lower oxidation state (Serpone., 2006). The loss of O atoms creates the formation of color center due to the charge trapped in cavity (V_o_) left behind (reaction 3). Such the impacted effects on visible-light driven reactivity for color centers are even more important than the narrowing band gap caused by doping Bjelajac et al. (2018).

**FIGURE 10:**
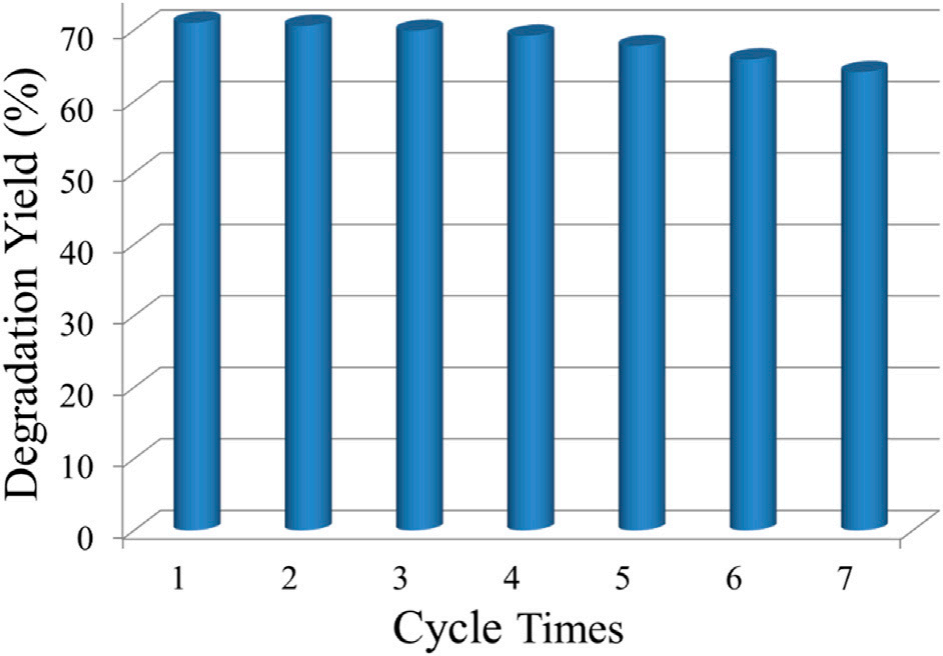
Degradation yield of Ti_0.8_Sn_0.2_O_2_ mixed solution after seven cycles.

For the improved spectral response in the visible region, the Ti_0.8_Sn_0.2_O_2_ solution also shows relatively stable photodegradation performance, as shown in Figure 10. The preformed powder is centrifuged and collected after every irradiation cycle and subsequently dispersed in a fresh RhB aqueous solution without any further purification. Compare with the first cycle, the photocatalyst displays a less than 10% decrease in activity after seven cycle times, endowing a cycling stability for solution with a mixed phase.

## 4 Conclusion

In summary, a facile and effective aerosol-spray strategy is developed to prepare high-performance photocatalyst by fabricating mesoporous Ti_1-x_Sn_x_O_2_ (0 < x < 1) solid solution. Ti_1-x_Sn_x_O_2_ nanocrystals with various Sn-doped contents are self-assembled into mesoporous spheres provide effective transport of charge carrier. As-prepared hierarchical architecture shows several advantages as a visible-light photocatalyst. First, mesoporous Ti_1-x_Sn_x_O_2_ spheres enhance the advantageous features of conventional titania and tin oxides. Second, heterostructure-based titania compounds further enhance photoinduced surface redox reactions and kinetic process in the visible region. Third, with increasing Sn-doped concentration, the mechanism between the anatase-rutile phase transition of Ti_1-x_Sn_x_O_2_ compounds and photocatalytic activity is further revealed. Therefore, as-prepared mesoporous Ti_1-x_Sn_x_O_2_ solid solution can effectively enhanced charge separation, and accelerated proton mass transfer. This demonstration of creating solid solutions offers a viable, cost-efficient method for the photocatalytic degradation of RhB, and this prototype has the potential to inspire the development of novel photocatalysts, such as splitting water, reducing CO_2_, and fixing N_2_.

## Data Availability

The original contributions presented in the study are included in the article/supplementary material, further inquiries can be directed to the corresponding author.
